# The PDE4 inhibitor tanimilast shows distinct immunomodulatory properties associated with a type 2 endotype and CD141 upregulation

**DOI:** 10.1186/s12967-022-03402-x

**Published:** 2022-05-10

**Authors:** Hoang Oanh Nguyen, Valentina Salvi, Laura Tiberio, Fabrizio Facchinetti, Mirco Govoni, Gino Villetti, Maurizio Civelli, Ilaria Barbazza, Carolina Gaudenzi, Mauro Passari, Tiziana Schioppa, Francesca Sozio, Annalisa Del Prete, Silvano Sozzani, Daniela Bosisio

**Affiliations:** 1 Department of Molecular and Translational Medicine, University of Brescia, Brescia, Italy; 2 Department of Experimental Pharmacology and Translational Science, Corporate Pre-Clinical R&D, Chiesi Farmaceutici S.p.A., Parma, Italy; 3 Global Clinical Development, Chiesi Farmaceutici S.p.A., Parma, Italy; 4 Laboratory Affiliated to Istituto Pasteur Italia-Fondazione Cenci Bolognetti, Department of Molecular Medicine, Sapienza University of Rome, Rome, Italy; 5 IRCCS Neuromed, Pozzilli, IS Italy

**Keywords:** CD141, Thrombomodulin, BDCA-3, cAMP, IL-13, Type 2 responses, Immune regulation

## Abstract

**Background:**

Tanimilast is a novel and selective inhaled inhibitor of phosphodiesterase-4 in advanced clinical development for chronic obstructive pulmonary disease (COPD). Tanimilast is known to exert prominent anti-inflammatory activity when tested in preclinical experimental models as well as in human clinical studies. Recently, we have demonstrated that it also finely tunes, rather than suppressing, the cytokine network secreted by activated dendritic cells (DCs). This study was designed to characterize the effects of tanimilast on T-cell polarizing properties of DCs and to investigate additional functional and phenotypical features induced by tanimilast.

**Methods:**

DCs at day 6 of culture were stimulated with LPS in the presence or absence of tanimilast or the control drug budesonide. After 24 h, DCs were analyzed for the expression of surface markers of maturation and activation by flow cytometry and cocultured with T cells to investigate cell proliferation and activation/polarization. The regulation of type 2-skewing mediators was investigated by real-time PCR in DCs and compared to results obtained in vivo in a randomized placebo-controlled trial on COPD patients treated with tanimilast.

**Results:**

Our results show that both tanimilast and budesonide reduced the production of the immunostimulatory cytokine IFN-γ by CD4^+^ T cells. However, the two drugs acted at different levels since budesonide mainly blocked T cell proliferation, while tanimilast skewed T cells towards a Th2 phenotype without affecting cell proliferation. In addition, only DCs matured in the presence of tanimilast displayed increased CD86/CD80 ratio and CD141 expression, which correlated with Th2 T cell induction and dead cell uptake respectively. These cells also upregulated cAMP-dependent immunosuppressive molecules such as IDO1, TSP1, VEGF-A and Amphiregulin. Notably, the translational value of these data was confirmed by the finding that these same genes were upregulated also in sputum cells of COPD patients treated with tanimilast as add-on to inhaled glucocorticoids and bronchodilators.

**Conclusion:**

Taken together, these findings demonstrate distinct immunomodulatory properties of tanimilast associated with a type 2 endotype and CD141 upregulation in DCs and provide a mechanistic rationale for the administration of tanimilast on top of inhaled corticosteroids.

## Introduction

Cyclic adenosine monophosphate (cAMP) is a ubiquitous second messenger that regulates numerous cellular functions including a vast array of immune and inflammatory processes. In particular, the elevation of intracellular cAMP reduces the production of pro-inflammatory mediators and favors the development of an anti-inflammatory environment [1]. Since the level of cAMP is critically regulated by phosphodiesterases (PDEs), these enzymes have recently been spotted as therapeutic targets in several inflammatory conditions [2]. As of now, there are eleven distinct PDE families (PDE1-11), responsible for nucleotide degradation (cAMP and/or cGMP). Among them, the PDE4 family, comprising PDE4A, PDE4B, PDE4C and PDE4D, is the main player in controlling cAMP hydrolysis in inflammatory and immune cells [3], so promoting the production of pro-inflammatory cytokines and lipid mediators [4]. Inhibition of PDE4 has been shown not only to shift the anti-inflammatory/pro-inflammatory balance but also to ameliorate pulmonary function and symptoms in patients with chronic respiratory diseases [5].

Tanimilast (international non-proprietary name of CHF6001) is a novel and potent selective inhaled PDE4 inhibitor developed for the treatment of asthma and chronic obstructive pulmonary disease (COPD) [6]. Tanimilast, that showed overlapping activity with roflumilast (another approved PDE4 inhibitor) in a previous extensive comparison performed by these authors [7], has currently completed phase IIa [8, 9] and phase IIb [9] clinical development showing promising results in different target indications [10, 11]. A global phase III development is undergoing in COPD patients with a chronic bronchitis phenotype and a history of exacerbations, as an add-on to triple therapy (inhaled glucocorticoid, long-acting β2-agonist, and long-acting muscarinic-receptor antagonist) [12, 13]. In this population, tanimilast showed a broad anti-inflammatory profile in the lung associated with limited systemic effects [14]. Indeed, tanimilast optimization for inhaled administration allows its high retention in the lung along with low systemic exposure [15], thus reducing systemic adverse events frequently reported with known oral PDE4 inhibitors [6]. The anti-inflammatory properties of tanimilast were characterized in several in vitro and in vivo models that demonstrated its ability to modulate a wide range of immune cells which play critical roles in many pathological conditions [16, 17]. In this regard, our previous publication described the capacity of tanimilast to modulate inflammation by down-regulating multiple pro-inflammatory mediators, especially those inducing Th1/Th17 responses and CD8^+^ T cell activation [18], secreted by activated human dendritic cells (DCs).

DCs are innate immune cells specialized in both cytokine production and antigen presentation to T lymphocytes. As such, DCs possess the capability to dictate the features of the arising adaptive immune response. For example, DCs may promote Th1 differentiation and CD8^+^ T cell cytotoxicity via IL-12 secretion or expand Th17 effectors upon the combined secretion of IL-23, IL-6 and IL-1β, to protect from intracellular and extracellular pathogens respectively. However, excessive T cell activation and polarization paves the way to the development of chronic inflammatory and autoimmune diseases [19, 20]. Notably, T cell activation is one of the main biological processes activated in COPD [21], together with Th1/Th17 skewed T-cell responses, CD8^+^ T cell activation and continuous inflammation with neutrophil and macrophage infiltration [22, 23]. Therefore, DCs are a relevant pharmacological targets to selectively modulate the T effector response in COPD [24].

The aim of this study was to thoroughly characterize the functions and the phenotype of activated human DCs in response to tanimilast as compared to budesonide, an inhaled corticosteroid commonly utilized as anti-inflammatory agent in COPD, and to validate these findings in vivo in sputum cells of COPD patients treated with tanimilast on top of inhaled corticosteroids.

## Materials and methods

### Cell preparation and culture

Buffy coats from blood donations of 3 to 4 (as specified in each Figure and legend) anonymous healthy donors were obtained and preserved by the Centro Trasfusionale, Spedali Civili of Brescia according to the Italian law concerning blood component preparation and analysis. Peripheral blood mononuclear cells (PBMC) were obtained by density gradient centrifugation and monocytes were subsequently purified by immunomagnetic separation using anti CD14-conjugated magnetic microbeads (Miltenyi Biotec) according to the manufacture’s protocol and as previously published [21]. Briefly, monocytes were cultured for 6 days in tissue culture plates in complete medium RPMI 1640 supplemented with 10% heat-inactivated, endotoxin free FBS, 2 mM l-glutamine, penicillin and streptomycin (all from Gibco, Thermo Fisher Scientific) in the presence of 50 ng/ml GM-CSF and 20 ng/ml IL-4 (Miltenyi Biotec). The maturation of moDCs was induced by incubation with 100 ng/ml LPS (*Escherichia coli* 055: B5, Sigma-Aldrich), heat-killed *E. coli* (moDC/bacteria 1:10, InvivoGen), 1 μg/ml R848 (InvivoGen), 25 μg/ml β-glucan (Sigma-Aldrich) for 24 h. Where indicated, cells were pretreated for 1 h with tanimilast or budesonide (all from Chiesi Pharmaceuticals), both at 10^−7^ M, a concentration previously characterized as effective and non-toxic in moDCs [18]. Untouched peripheral blood cDC1 and cDC2 (cDCs) were obtained from PBMC after immunomagnetic separation with the CD1c (BDCA-1) Dendritic Cell Isolation Kit (Miltenyi Biotec) and CD141 (BDCA-3) MicroBead Kit, respectively. The maturation process was conducted in RPMI containing 2% FBS and supplemented with 0.01% DMSO to avoid the sequestration of tanimilast by serum proteins.

### Flow cytometry

DCs were stained with the following antibodies from Miltenyi Biotec and Biolegend: Vioblue-conjugated anti-human CD86 (clone FM95; dilution 1/100), PE-Vio770-conjugated anti-human CD80 (clone 2D10; 1/20), APC-Vio770-conjugated anti-human HLA-ABC (MHC-I, clone REA230; 1/200), PE-conjugated anti-human PD-L1 (clone 29E.2A3; 1/60), APC-conjugated anti-human ILT-3 (clone REA141; 1/20), PE or APC-conjugated anti-human CD141 (clone AD5-14H12, 1/100), PE-Vio770-conjugated anti-human CD1c (clone REA649; 1/50). Cell viability was assessed by LIVE/DEAD staining according to the manufacturer’s instruction (Molecular Probes, Thermo Fisher Scientific). Samples were read on a MACSQuant Analyzer (Miltenyi Biotec) and analysed with FlowJo (Tree Star Inc). Response definition criteria were defined post-hoc. Raw data can be provided per request.

### Real-time PCR

RNA was extracted using TRIzol reagent, treated with DNAse according to the manufacturer’s instructions and reverse transcription performed using random hexamers and MMLV RT (all from Thermo Fisher Scientific). The SsoAdvanced Universal SYBR Green Supermix (Bio-Rad Laboratories) was used according to the manufacturer’s instructions. Reactions were run in triplicate on a StepOne Plus Real-Time PCR System (Applied Biosystems) and analyzed by the StepOne Plus Software (Version 2.3, Applied Biosystems). Sequences of gene-specific primers were listed in Table 1. Gene expression was normalized based on HPRT mRNA content.

**Table 1:** Sequences of gene-specific primers used for RT-PCR

Primers	Forward	Reverse
hHPRT	5′-CCAGTCAACAGGGGACATAAA-3′	5′-CACAATCAAGACATTCTTTCCAGT-3′
hIL12p40	5′-CTAAGCCATTCGCTCCTGCTG-3′	5′-CTTGGCCTCGCATCTTAGAAAGG-3′
hIRF8	5′-TGGCTGATCGAGCAGATTGACAGT-3′	5′-AAGGGATCCGGAACATGCTCTTCT-3′
hIDO1	5′-ACCAGCTCACAGGAACTTCCTG-3′	5′-CCTGTGTGAAAGCTCTGGTCTC-3′
hTSP1	5′-CAATGCCACAGTTCCTGATG-3′	5′-TGGAGACCAGCCATCGTC-3′
hVEGF-A	5′-ATGCGGATCAAACCTCACCAA-3′	5′-TGTCTTGCTCTATCTTTCTTTGG-3′
hAREG	5′-CGGAGAATGCAAATATATAGAGCAC-3′	5′-CACCGAAATATTCTTGCTGACA-3′

### T cell proliferation assay

Allogenic naïve CD4^+^ T cells and CD8^+^ T cells were isolated from buffycoats using the naïve CD4^+^ T cell Isolation kit II (Miltenyi Biotec) and CD8^+^ T cell Isolation kit (Miltenyi Biotec), respectively. Purified T cells were labeled with CellTrace-CFSE (Molecular Probes, Thermo Fisher Scientific) at a final concentration of 5 μM. Subsequently, T cells (6 × 10^4^ cells/well) were co-cultured with graded numbers of allogeneic moDCs in 96-well round-bottom culture plates in complete RPMI medium. After 6 days, alloreactive T cell proliferation was assessed by measuring the loss of the dye CellTrace-CFSE upon cell division using flow cytometry. Positive controls of T cell proliferation were routinely performed using IL-2 plus PHA. Response definition criteria were defined post-hoc. Dead cells were excluded by LIVE/DEAD staining according to the manufacturer’s instruction. Raw data can be provided per request.

### Analysis of T cell cytokine production

After 6 days of co-culture, CD4^+^ and CD8^+^ T cells were restimulated with 200 nM PMA (Sigma-Aldrich) plus 1 μg/ml of ionomycin (Sigma) for 4.5 h. Brefeldin A (5 μg/ml, Sigma-Aldrich) was added during the last 2 h. For intracellular cytokine production, cells were fixed and permeabilized with Inside Stain kit (Miltenyi Biotec) and stained with FITC-conjugated anti-IFN-γ (clone 45-15, Miltenyi Biotec), APC-conjugated anti-IL-13 (clone OES10-5A2, Biolegend), APC-conjugated anti GrB (clone REA226) following the manufacturer’s recommendations. Response definition criteria were defined post-hoc. Raw data can be provided per request.

### Dead cell uptake

Cryopreserved autologous PBMCs were thawed, labeled with CFSE and then heat-killed (90 °C for 30 min) as previously described [25]. Dead cells were added to moDCs at a 1:1 ratio for 2 h at 37 °C. For flow cytometric analysis, moDCs were stained for HLA-DR (MHC-II), and the percentage of dead cell uptake defined as MHC-II^+^CFSE^+^ cells was measured.

### Clinical investigation

Investigations were performed on data obtained from patients enrolled in a multicentre, three-way, placebo-controlled, double-blind crossover study [9] who received 1-month treatment with two therapeutic doses of tanimilast (800 or 1600 μg twice daily) or matching placebo; ClinicalTrials.gov (NCT03004417). Patients had to be on stable treatment with inhaled triple therapy (ICS/LABA/LAMA) for at least 2 months prior to enrolment and have a post-bronchodilator ratio of forced expiratory volume in 1 s (FEV1) to forced vital capacity ratio < 0.70 and FEV1 30–70% predicted, and a history of chronic bronchitis. Induced sputum samples had to have a viability factor of at least 70% and epithelial cells lower than 30%. Samples were collected pre-dose and end of treatment and processed for microarray mRNA assessments as previously described [14]. The study was approved by independent ethics committees for each institution. All patients provided written informed consent prior to study start. Gene expression raw data were deposited in NCBI’s Gene Expression Omnibus and are accessible through GEO Series accession number GSE133513 (https://www.ncbi.nlm.nih.gov/geo/query/acc.cgi?acc=GSE133513).

### Statistical analysis

Sample group normality was confirmed by Shapiro–Wilk test before application of parametric statistical analysis. Statistical significance among the experimental groups was determined using one-way ANOVA with Dunnet’s post-hoc test (GraphPad Prism 7, GraphPad Software) as indicated in each figure legend.

In patients, an ANCOVA model was fitted to identify probe sets responding differentially to treatment, with change from pre-dose to post-dose expression as dependent variable, and subject, period, treatment and pre-dose expression as independent variables. Data analysis was performed in R version 4.0 (R Core Team, Vienna, Austria, 2020).

P < 0.05 was considered significant.

## Results

### Tanimilast impaired the Th1-promoting capacity of moDCs activated by LPS (LPS-moDCs)

**Fig. 1:**
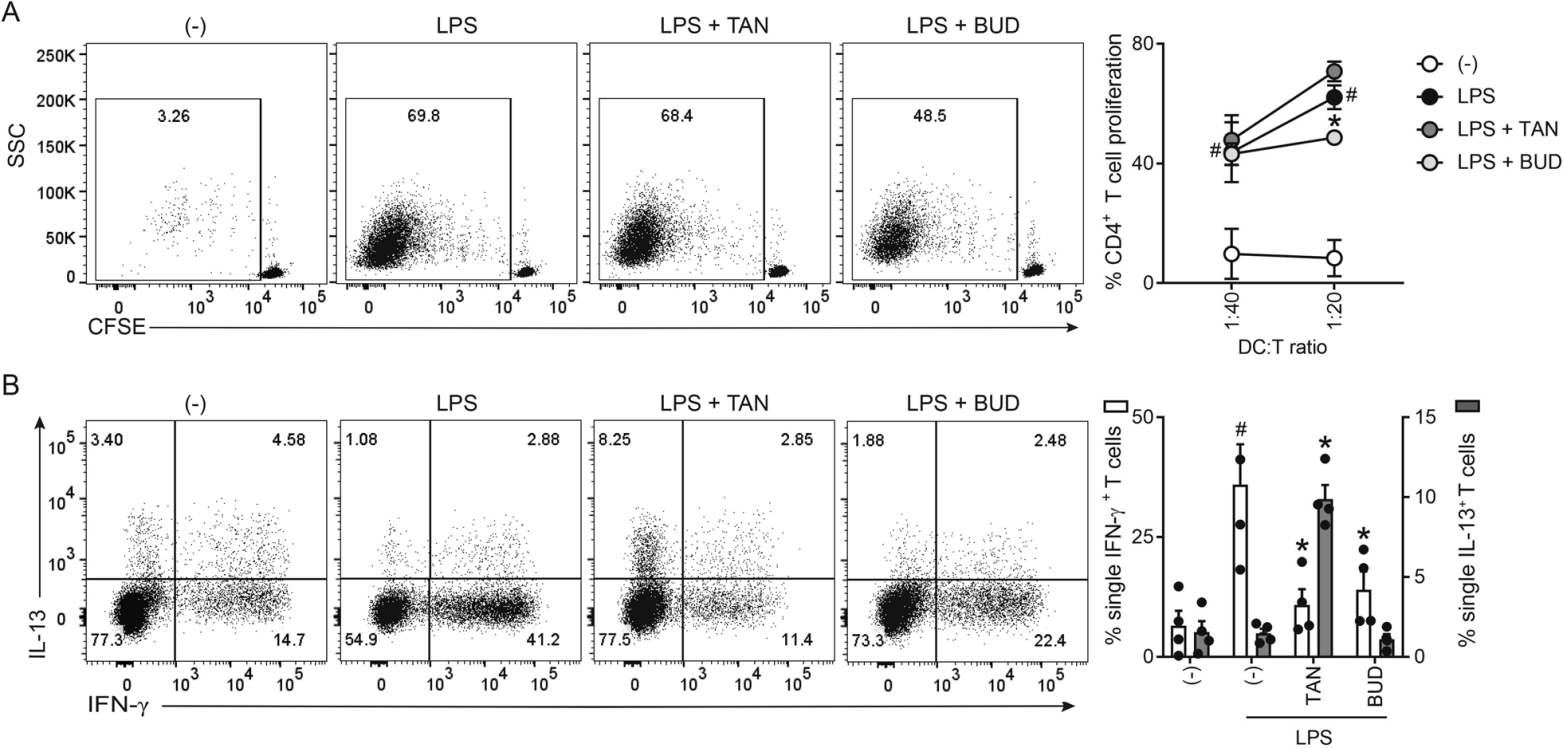
Effect of tanimilast on CD4^+^ T cell activation by LPS-moDCs. **A** moDCs were treated or not (-) with tanimilast (TAN) or budesonide (BUD) (both at 10^−7^ M) for 1 h before stimulation with LPS. After 24 h, moDCs were collected and co-cultured with CFSE-stained allogenic CD4^+^ T cells for 6 days. Alloreactive T cell proliferation was assessed by measuring CellTrace-CFSE dye loss by flow cytometry. Left, dot plot from one representative experiment (1:20 ratio). Right, line graph from three independent experiments with the indicated DC:T cell ratio. Data are expressed as mean ± SEM (n = 3) of the percentage of proliferating CD4^+^ T cells. **B** moDCs treated as described in **A** were incubated with T cells for 6 days. Intracellular IFN-γ and IL-13 were evaluated by FACS analysis. Left, dot plot from one representative experiment (1:20 ratio). Right, bar graphs from four independent experiments. Data are expressed as mean ± SEM (n =  4) of single IFN-γ-(left Y axis) and IL-13-(right Y axis) producing T cells. **A**, **B** ^#^P < 0.05 versus (-) and *P < 0.05 versus LPS by one-way ANOVA with Dunnett’s post-hoc test

We previously demonstrated that tanimilast diminished the Th1/Th17 polarizing potential of LPS-moDCs by decreasing the secretion of cytokines such as IL-12, IL-23 and IL-1β, while increasing the Th2-recruiting chemokine CCL22 [18]. Here, we set up allogeneic co-culture experiments to investigate the functional outcome of these modulations in terms of T cell stimulatory and polarizing capacity. Budesonide, an inhaled corticosteroid commonly prescribed in asthma and COPD, was used as a comparison [26]. moDCs matured by LPS exposure in the presence of tanimilast (TAN-LPS-moDCs) or budesonide (BUD-LPS-moDCs) were co-cultured with naïve CD4^+^ T cells for 6 days. The proliferative capacity of naïve CD4^+^ T cells was not affected by tanimilast, whereas budesonide significantly reduced the percentage of proliferating cells (Fig. 1A). Both drugs blunted the prominent Th1 response elicited by LPS-moDCs, as evaluated in terms of decreased percentage of IFN-γ producing T cells by intracellular staining (Fig. 1B, left axis). However, only TAN-LPS-moDCs enhanced the development of T cells producing IL-13, which characterizes a Th2 response (Fig. 1B, right axis). In the present experimental conditions, both CD25^+^Fox3p^+^ T cells and IL-17^+^ cells were barely detectable and not modulated by the drugs (data not shown). Control experiments demonstrated that tanimilast did not induce IL-13^+^ T cells when administered to immature moDCs (data not shown).

### *Tanimilast suppressed the activation of CD8*^+^*T cells by LPS-moDCs*

**Fig. 2:**
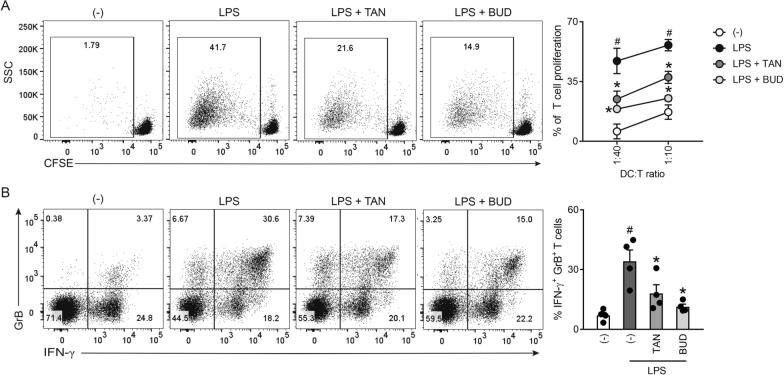
Effect of tanimilast on CD8^+^ T cell activation by LPS-moDCs. **A** moDCs were treated or not (-) with tanimilast (TAN) or budesonide (BUD) (both at 10^−7^ M) for 1 h before stimulation with LPS. After 24 h, moDCs were collected and co-cultured with CFSE-stained allogenic CD8^+^ T cells for 6 days. Alloreactive T cell proliferation was assessed by measuring CellTrace-CFSE dye loss by flow cytometry. Left, dot plot from one representative experiment (1:40 ratio). Right, line graph from three independent experiments with the indicated DC:T cell ratio. Data are expressed as mean ± SEM (n = 3) of the percentage of proliferating CD8^+^ T cells. **B** moDCs treated as described in **A** were incubated with T cells for 6 days. Intracellular IFN-γ and Granzyme B (GrB) were evaluated by FACS analysis. Left, dot plot from one representative experiment (1:40 ratio). Right, bar graphs from four independent experiments. Data are expressed as mean ± SEM (n = 4) of IFN-γ^+^ GrB^+^ producing T cells. **A**, **B** ^#^P < 0.05 versus (-) and *P < 0.05 versus LPS by one-way ANOVA with Dunnett’s post-hoc test

Co-culture experiments were also performed using CD8^+^ T cells as responders. At difference with CD4^+^ T effectors, both tanimilast and budesonide similarly attenuated the proliferation of CD8^+^ T cells induced by LPS-moDCs (Fig. 2A). Also, both drugs substantially decreased the percentage of cells producing IFN-γ and Granzyme B, two key effector molecules of activated CD8^+^ T cells (Fig. 2B).

Taken together, these results indicate that both tanimilast and budesonide modulate the T activating potential of LPS-moDCs by impairing Th1 induction and reducing the cytotoxic potential of CD8^+^ T cells. Unlike budesonide, tanimilast did not affect the capacity of LPS-moDCs to stimulate CD4^+^ T cell proliferation and favored the development of a Th2-oriented immune profile.

### Tanimilast regulated a broad panel of genes involved in T cell immunosuppression

**Fig. 3:**
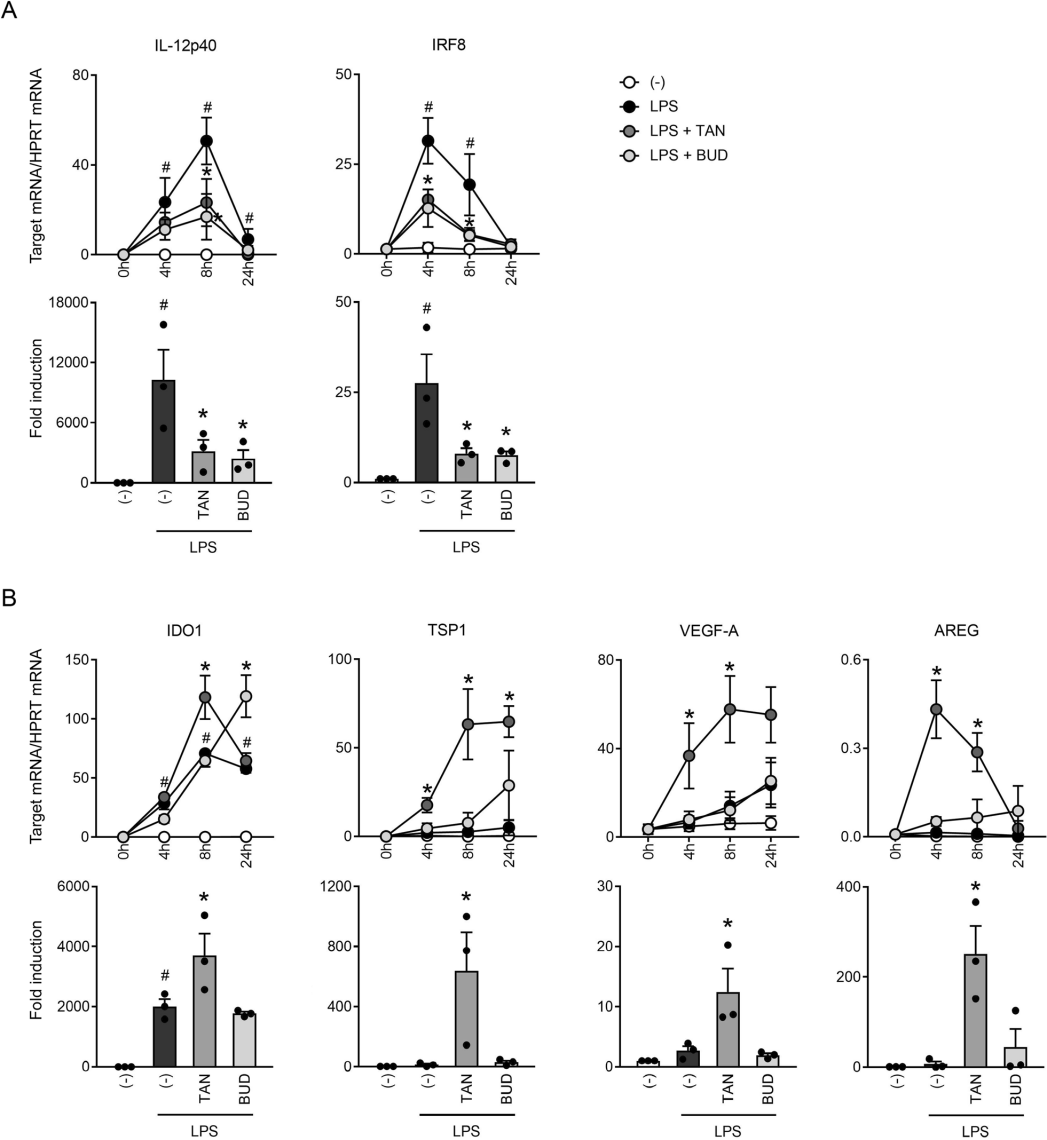
Effect of tanimilast on mRNA expression of genes involved in regulation of inflammation. moDCs pre-treated or not with tanimilast (TAN) or budesonide (BUD) (both at 10^−7^ M) and subsequently stimulated with LPS for the indicated time points. mRNA expression levels were evaluated by Real-Time PCR. Downregulated (**A**) and upregulated (**B**) gene mRNAs by drug pre-treatment are shown. Pre-treatment did not modify the mRNA levels of any of these genes in unstimulated DCs (-) (not shown). Data are expressed as mean ± SEM (n = 3) of 2^−ΔCt^ relative to housekeeping mRNA (HPRT) (upper panels) as well as mean ± SEM (n = 3) of 2^−ΔΔCt^ relative to (-) (fold induction at 8-h time-point, lower panels); ^#^P < 0.05 versus (-) or *P < 0.05 versus LPS by one-way ANOVA with Dunnett’s post-hoc test

Based on the findings above, we set out to better characterize the immunomodulatory potential of TAN-LPS-moDCs. To this extent, immature and mature moDCs pre-treated with tanimilast or budesonide were analyzed for the expression of steady-state mRNAs encoding for proteins associated with immune activation or tolerance as depicted in Fig. 3. In line with the marked reduction of the protein levels described in our previous work [18], IL-12p40 mRNA was considerably downregulated in response to both drugs (Fig. 3A, left). Both tanimilast and budesonide also remarkably inhibited the expression of IRF8, a transcription factor involved in IL-12 production [27] (Fig. 3A, right). Notably, the downregulation of IL12p40 mRNA expression was also shown in sputum cell of COPD patients when tanimilast was administered on top of stable doses of corticosteroids, while IRF8 showed a tendency towards a decrease without reaching statistical significance (Table 2).

**Table 2:** Preselected c-AMP dependent genes found to be significantly differentially expressed in sputum cells of COPD patients after treatment with tanimilast 1600 μg/day or 3200 μg/day relative to standard of care [inhaled corticosteroid (ICS) plus dual bronchodilators (LABA + LAMA)]

ID	Gene symbol	Gene title	Entrez gene	Fold change (tanimilast 1600 μg/day vs. placebo)	p value (tanimilast 1600 μg/day vs. placebo)	Fold change (tanimilast 3200 μg/day vs. placebo)	p value (tanimilast 3200 μg/day vs. placebo)
207901_at	IL12B	Interleukin 12B	3593	− 2.23	4.37E−06*	− 1.86	2.83E−04*
204057_at	IRF8	Interferon regulatory factor 8	3394	− 0.12 (ns)	0.12	− 1.07	0.20 (ns)
210029_at	IDO1	Indoleamine 2,3-dioxygenase 1	3620	−1.24	0.2538 (ns)	1.25	0.2207 (ns)
201108_s_at	THBS1	Thrombospondin 1 (TSP1)	7057	1.23	4.44E−02*	1.54	3.04E−05*
212171_x_at	VEGFA	Vascular endothelial growth factor A	7422	1.19	1.44E−02*	1.23	3.39E−03*
1557285_at	AREG	Amphiregulin	374	2.05	1.86E−04*	2.89	1.31E−07*
203888_at	THBD	Thrombomodulin (CD141)	7056	1.12	5.15E−03*	1.12	4.14E−03

*P < 0.05

By contrast, tanimilast upregulated the levels of the suppressive mediators IDO1, TSP1, VEGF-A and AREG (Fig. 3B). BUD-LPS-moDCs displayed a delayed IDO upregulation and low or no induction of the other mRNAs, which is consistent with the previously described dependence of these genes’ expression on intracellular cAMP levels [28, 29]. In COPD patients, tanimilast exerted an add-on effect to inhaled corticosteroids with upregulation of the expression of TSP1, VEGF-A and AREG in sputum cells of the lung, while IDO1 modulation did not reach statistical significance (Table 2).

These results highlight a different ability of TAN-LPS-moDCs and BUD-LPS-moDCs in the upregulation of multiple immunosuppressive molecules.

### Tanimilast modulated the phenotype of LPS-moDCs

**Fig. 4:**
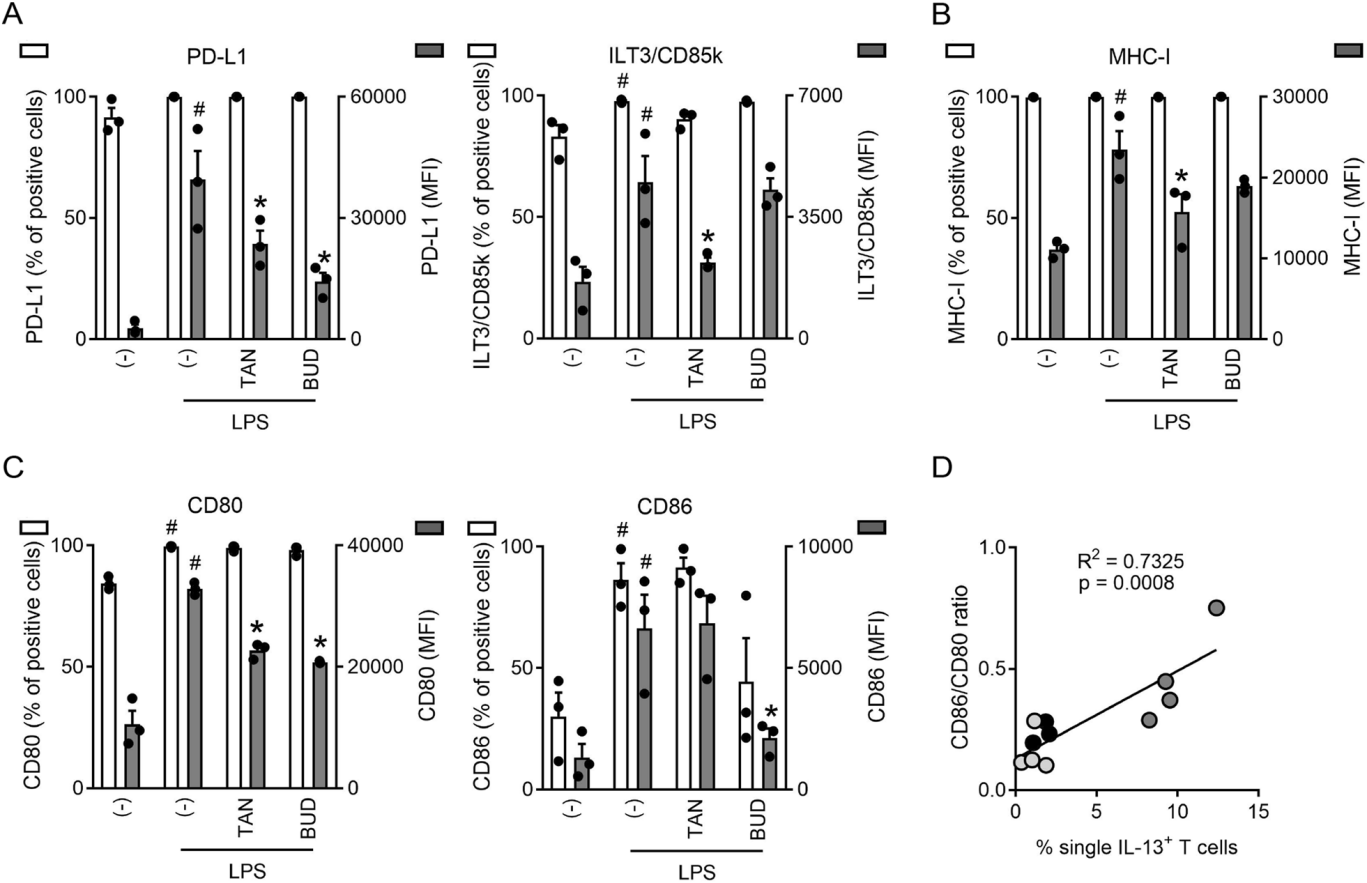
Effect of tanimilast on LPS-moDC phenotype. moDCs were pre-treated or not (-) with either tanimilast (TAN) or budesonide (BUD) (both at 10^−7^ M) for 1 h and subsequently stimulated or not with LPS for 24 h. The surface expression of regulatory markers PD-L1, ILT3/CD85k (**A**) and of maturation markers MHC-I (**B**), CD80, CD86 (**C**) were evaluated by FACS analysis. Data are expressed as the mean ± SEM (n = 3) of the percentage of positive cells (left y axis) and of the Median Fluorescence Intensity (MFI) (right y axis). (**A**–**C**) ^#^P < 0.05 versus (-) and *P < 0.05 versus LPS by one-way ANOVA with Dunnett’s post-hoc test. (**D**) Correlation between CD86/CD80 ratios and % of single IL-13^+^ T cells induced by LPS-moDCs (black dots), TAN-LPS-moDCs (dark grey dots) or BUD-LPS-moDCs (light grey dots) from 3/4 donors (R^2^ = 0.7325; Spearman r = 0.8559, ***P = 0.0008)

Next, we investigated if TAN-LPS-moDCs differ from LPS-moDCs and BUD-LPS-moDCs in terms of phenotypic markers, with a particular focus on surface molecules that play a role in T cell activation and immunoregulation, such as PD-L1 and ILT3/CD85k. We also investigated the expression of Ox40L, a positive signal for Th2 differentiation [30], but this was undetectable in our system in all conditions (data not shown). Figure 4A shows that both PD-L1 and ILT3/CD85k were constitutively expressed by all resting moDCs (left y axis) at low levels (right y axis), that were potently increased by LPS stimulation (right y axis). Both tanimilast and budesonide decreased the LPS-induced expression of PD-L1 (right y axis), possibly because of the previously described suppression of LPS-induced inflammatory cytokines [18, 31]. By contrast, tanimilast but not budesonide decreased ILT3/CD85k.

When checking the regulation of classical maturation markers, we found that TAN-LPS-moDCs showed reduced expression of MHC-I (Fig. 4B). Tanimilast also reduced the expression of the costimulatory molecule CD80 (Fig. 4C, left panel), while CD86 (Fig. 4C, right panel) was not affected. Budesonide behaved similarly to tanimilast (Fig. 4B, C, left panel), with the notable exception that it also induced a significant decrease of CD86 (Fig. 4C, right panel). The counter-regulation of CD80 and CD86 determined a higher CD86/CD80 ratio in TAN-LPS-moDCs as compared to BUD-LPS-moDCs and LPS-moDCs, which we found to be positively correlated with the induction of IL-13^+^ T cells (Fig. 4D).

We also investigated the expression of CCR2 and CCR6, two chemokine receptors responsible for DC accumulation into the lung [32]. However, these remained unaltered and undetectable, respectively, in all our experimental conditions (data not shown). When administered to immature moDCs, both tanimilast and budesonide did not modify the basal levels of any of the analyzed markers (data not shown).

### CD141 expression marks DCs matured in the presence of tanimilast

**Fig. 5:**
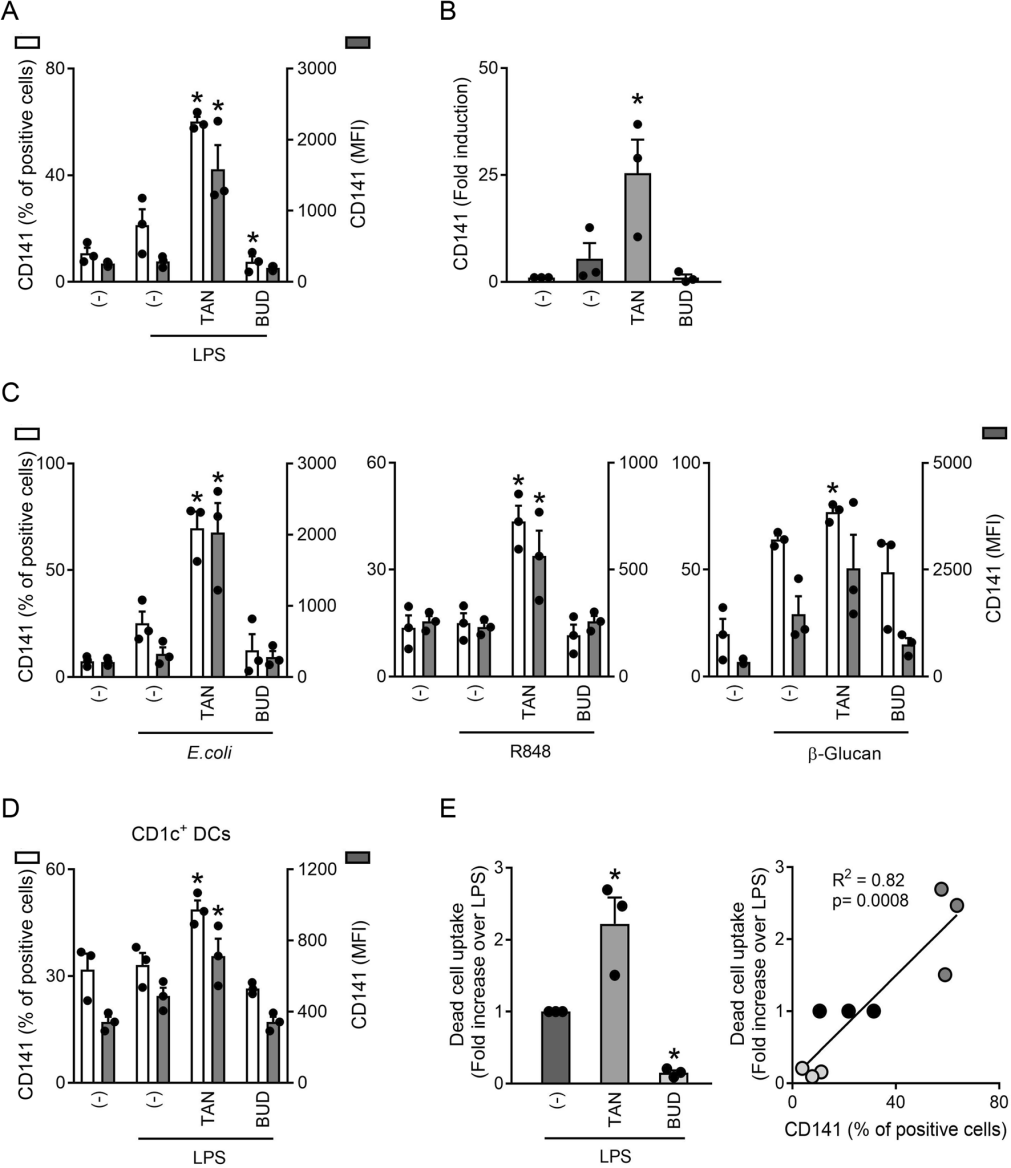
Effect of tanimilast on CD141 (Thrombomodulin/BDCA3) expression in DCs. **A**, **B** moDCs were pre-treated or not (-) with tanimilast (TAN) or budesonide (BUD) (both at 10^−7^ M) for 1 h and subsequently stimulated with LPS for 24 h. The expression of CD141 was evaluated by FACS analysis (**A**) and by Real time PCR (**B**). Data are expressed as mean ± SEM (n = 3) of the percentage of positive cells (left y axis) and of the Median Fluorescence Intensity (MFI) (right y axis) (**A**) and of 2^−ΔΔCt^ relative to (-) (fold induction at 24-h time-point) (**B**). moDCs (**C**) or CD1c^+^ DCs (**D**) were treated as described in **A** and subsequently stimulated or not with *E. coli*, R848, β-glucan (**C**) or LPS (**D**) for 24 h. The surface expression of CD141 were evaluated by FACS analysis. Data are expressed as the mean ± SEM (n = 3) of the percentage of positive cells (left y axis) and of the Median Fluorescence Intensity (MFI) (right y axis). ^#^P < 0.05 versus (-) and *P < 0.05 versus LPS (**A**, **B**, **D**) or *E. coli*, R848, β-glucan (**C**) by one-way ANOVA with Dunnett’s post-hoc test. **E** moDCs treated as described in **A** were labeled with MHC-II and then co-cultured with heat-killed autologous CFSE^+^ PBMC at ratio 1:1 for 2 h. Dead cells uptake was defined as the percentage of MHC-II^+^CFSE^+^ cells. Left panel represents the fold increase of dead cell uptake upon LPS stimulation. Data are expressed as the mean ± SEM (n = 3) of the fold increase. Right panel represents the correlation between the percentage of CD141^+^ cells and dead cell uptake by LPS-moDCs (black dots), TAN-LPS-moDCs (dark grey dots) and BUD-LPS-moDCs (light grey dots) from 3 donors (R^2^ = 0.82; Spearman r = 0.9055, ***P = 0.0008)

In the search for markers of TAN-LPS-moDCs, we serendipitously observed that these cells potently upregulated membrane CD141, also known as Thrombomodulin or BDCA-3, both in terms of percentage of positive cells and MFI (Fig. 5A), and also increased the expression of CD141 mRNA (Fig. 5B), while BUD-LPS-moDCs did not (Fig. 5A, B). Consistent with this, the expression of CD141 was also found to be upregulated in vivo in sputum cells of COPD patients, as an additive effect of tanimilast to inhaled corticosteroids (Table 2). The upregulation of CD141 by tanimilast was not restricted to moDCs activated by LPS since it could be reproduced when moDCs were matured using different proinflammatory agonists such as *E. coli*, R848 and β-glucan (Fig. 5C). Figure 5D demonstrates the ability of tanimilast to upregulate the expression of CD141 also in LPS-activated primary CD1c^+^ DCs.

CD141 is the distinctive marker of conventional DCs type 1 (cDC1s) [33], a subpopulation of primary DCs specialized in antigen cross-presentation following dead cell uptake [34] and also known to induce T cells that preferentially produce type 2 cytokines [35]. Thus, we asked if moDCs in the presence of tanimilast may acquire features of cDC1s. Indeed, tanimilast (but not budesonide) potentiated the capacity of LPS-moDCs to uptake dead cells (Fig. 5E, left panel), an effect that positively correlated with the percentage of CD141-expressing cells (Fig. 5E, right panel).

These results show that the expression of CD141 discriminates DCs matured in the presence of tanimilast as compared to budesonide and suggest that CD141 may represent a marker of newly described immunomodulatory DCs induced by tanimilast.

## Discussion

**Fig. 6:**
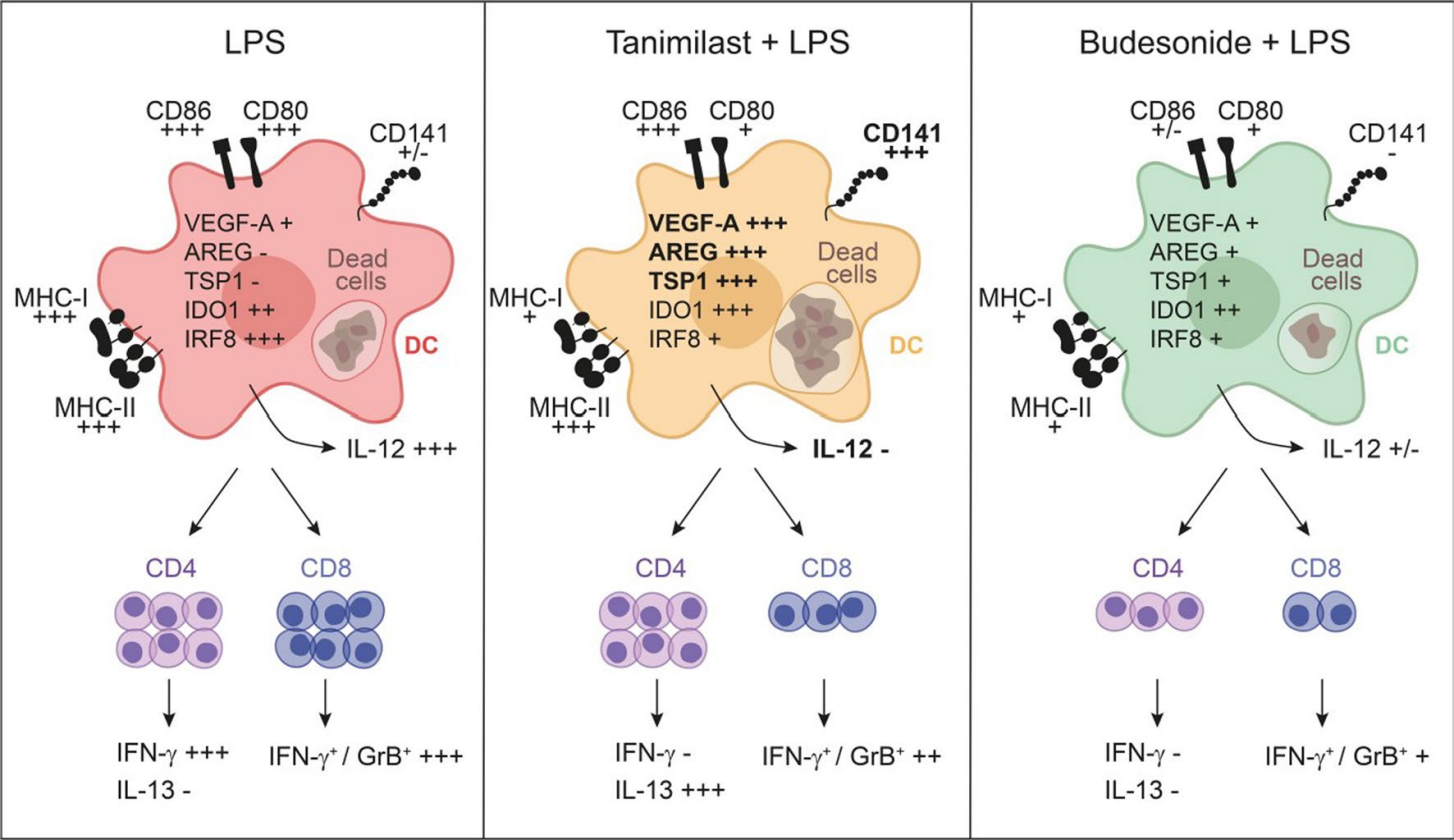
Schematic illustration of the effects of tanimilast and budesonide on LPS-moDC phenotype and function. DCs matured in the presence of tanimilast express distinct phenotypical and functional properties when compared to budesonide, characterized by the upregulation of CD141 expression and the acquisition of Th2-skewing properties. Bold lettering indicates that the upregulation of IL-12p40, TSP1, VEGF-A, AREG and CD141 was also observed in vivo in sputum cells of COPD patients treated with tanimilast on top of standard-of-care therapy

Tanimilast displays prominent anti-inflammatory properties in several cell-based models [7, 36] as well as in experimental rodent models of pulmonary inflammation [17] and in clinical settings [6]. In addition, we have previously demonstrated that it also finely tunes, rather than suppressing, the cytokine network produced by inflamed DCs, thus potentially modulating T cell polarization and adaptive effector functions [18, 37]. Here, we characterized TAN-LPS-moDCs as compared to BUD-LPS-moDCs as activators of CD4^+^ and CD8^+^ T cells. Both tanimilast and budesonide reduced the secretion of IFN-γ by activated T cells and inhibited CD8^+^ T cell proliferation and acquisition of the cytotoxic protease Granzyme B. However, only TAN-LPS-moDCs also induced the differentiation of IL-13^+^ CD4^+^ T cells, which indicates a type 2 oriented skewing of the immune response (Fig. 6).

Type 2 immunity was initially linked to host defence against helminths and, when occurring in response to environmental proteins, to allergy and anaphylaxis. A more current view, however, suggests that type 2 responses may play a broader role in immune surveillance at tissue barrier sites, repair responses, and the restoration of homeostasis [38]. Thus, the type 2-orienting property of tanimilast described in this work may further support its application to pathological conditions characterized by inflammation- and immune-mediated tissue damage such as asthma and COPD. Although the link between cAMP-elevating agents (such as tanimilast) and the establishment of type 2 responses has been previously described [39, 40], the present work details a number of mechanisms underlying the Th2-promoting properties of tanimilast. Of possible paramount importance is the reduction of IL-12 [18], the master Th1-promoting and Th-2 suppressing cytokine, observed both in in vitro and in vivo in sputum cells of COPD patients treated with tanimilast on top of inhaled corticosteroids. It is tempting to hypothesize that IL-12 suppression may correlate with the observed decrease of IRF8, a transcription factor known to positively regulate the p40 subunit of this cytokine [27, 41]. In addition, since increased IRF8 correlated with the development of chronic inflammatory diseases characterized by excessive Th1/Th17 response [42, 43], its reduction may play immunomodulatory roles also beyond IL-12 reduction. Tanimilast also upregulated the expression of IDO1, a marker of regulatory DCs involved in the suppression of Th1/Th17 mediated pathologic conditions [44, 45], as well as that of TSP1, VEGF-A and AREG that play roles both in inhibiting the activity of T cells [46–48] and in resolving inflammation [49–51] (Fig. 6). IDO and VEGF-A were also shown to act as direct Th2-orienting mediators in some conditions [52, 53], while VEGF protected against progression and severity of COPD. By contrast, its deficiency induced by cigarette smoke impaired lung alveolar structure [54]. Of note, human DCs are a well-known source of these factors, especially in contexts characterized by elevated intracellular levels of cAMP [28, 29, 46, 55]. Finally, we also propose a Th2-orienting role for the differentially regulated costimulatory molecules CD80 and CD86 (Fig. 6) by showing that the increased CD86/CD80 ratio induced by tanimilast strongly correlates with the induction of IL-13^+^ T cells, in line with previous findings describing the reduction of Th1 polarization upon decreased levels of CD80 and preservation of the Th2-associated molecule CD86 [56–58].

We also describe a number of other phenotypical changes induced by tanimilast. LPS-moDCs expressed high levels of PD-L1, which restrains the functions of activated T cells [59] and ILT3/CD85k, which negatively regulates DC activation in an autologous manner [60]. This increase was previously interpreted as a feedback loop to prevent excessive T cell responses, at least partially dependent on autologous cytokine secretion. Thus, both tanimilast and budesonide may counteract PD-L1 upregulation by suppressing LPS-induced inflammatory cytokines [18, 31]. By contrast, the downregulation of ILT3/CD85k observed with tanimilast, but not budesonide, may be related to the potent induction of COX2 by cAMP-elevating agents such as tanimilast (data not shown and [61]), which was previously shown to negatively regulate the expression of this marker [62]. The biological significance of such regulation, however, is difficult to predict based on our in vitro model. By contrast, the observed reduction of MHC-I well correlates with the decreased proliferation of CD8^+^ T cells described in Fig. 2, while the previously demonstrated untouched MHC-II [18] is consistent with unaffected CD4^+^ T cell proliferation. Of note, both MHC-I and CD80 are known to be regulated by intracellular cAMP levels [63, 64]. Taken together, our in vitro data depict TAN-LPS-moDCs as immunomodulatory cells with a distinct phenotype, also characterized by the specific upregulation of CD141. More specifically, we propose these cells as pro-resolving mediators that, by skewing strong Th1/Th17 immune activation towards a type 2 response, may help restore homeostasis at the site of injury [65].

Importantly, our in vitro findings were supported in vivo by data from a placebo controlled randomized trial in COPD patients treated with two doses of tanimilast and placebo on top of background standard-of-care therapy (inhaled corticosteroid plus bronchodilators) (Fig. 6, bold lettering). Notably, the doses, patient population and background therapy of this trial were the same of the currently ongoing phase III pivotal studies [12, 13]. In these conditions, the expression of TSP1, VEGF-A, AREG and CD141 genes in sputum cells was upregulated by both doses of tanimilast in comparison to placebo (standard-of-care), suggesting that the immunomodulatory effects of tanimilast observed in moDCs could be translated to a wider and more heterogeneous environment such as that of sputum cells of the airways of COPD patients. Moreover, being these effects observed on top of corticosteroids, our findings build a mechanistic rationale for the administration of tanimilast on top of triple therapy. In particular, COPD patients with prominent type 2 inflammatory endotype were shown to display a preferential response to inhaled corticosteroids [66]. Thus, tanimilast administered on top of corticosteroids could skew the immune response towards the type 2 endotype, which in turn is more responsive to corticosteroids. This may result in a more than additive effect of the combination in those patients characterized by prominent Th1/Th17 inflammatory features. This speculation is also supported by published data with roflumilast, an oral PDE4 inhibitor approved for the treatment of severe COPD, whose clinical effect size was shown to be greater when given in combination to inhaled corticosteroid [67].

In conclusion, our findings show distinct immunomodulatory properties of tanimilast when compared to budesonide, as highlighted by the differential regulation of type 2-orienting immune mediators. These features were also observed in vivo in sputum cells of COPD patients under stable treatment with corticosteroids. Taken together, these results provide a mechanistic rationale for the administration of tanimilast in COPD patients who are still symptomatic despite treatment with corticosteroid and bronchodilators.

## Data Availability

The original contributions presented in the study are included in the article/supplementary material. Further inquiries can be directed to the corresponding author.

## Abbreviations

BUD: Budesonide
cAMP: Cyclic adenosine monophosphate
COPD: Chronic obstructive pulmonary disease
DC: Dendritic cell
cDC: Conventional dendritic cell
moDC: Monocyte derived dendritic cell
ICS: Inhaled glucocorticoid
LABA: Long-acting β2-agonist
LAMA: Long-acting muscarinic-receptor antagonist
PDE: Phosphodiesterase
PBMC: Peripheral blood mononuclear cells
TAN: Tanimilast
